# Technology-enhanced learning in nursing education: a systematic review

**DOI:** 10.1186/s12909-026-10098-w

**Published:** 2026-08-11

**Authors:** Maha Besbes, Anaam Soloh, Mohammed Mustafa, Mohammed Elmadani, Osama Hamad, Godfrey Mbaabu Limungi, Orsolya Máté

**Affiliations:** 1 Doctoral School of Health Sciences, Faculty of Health Sciences, University of Pécs, Pécs, Hungary; 2 Institute of Emergency Care, Pedagogy of Health and Nursing Sciences, Faculty of Health Sciences, University of Pécs, Pécs, Hungary; 3 Ensana Thermal Margaret Island, Health Spa Hotel, Budapest, Hungary

**Keywords:** Nursing education, Artificial intelligence, Virtual reality, High-fidelity simulation, Gamification

## Abstract

**Background:**

Technology-enhanced learning (TEL), including artificial intelligence (AI), virtual reality (VR), high-fidelity simulation (HFS), and gamification, is increasingly used in nursing education to address limitations in clinical training and support the development of students’ clinical reasoning skills. This review aimed to synthesize current evidence on the educational outcomes associated with AI, VR, HFS, and gamification in nursing education, and to identify the factors that facilitate or hinder their successful implementation.

**Methods:**

A systematic review was conducted in accordance with PRISMA 2020 and registered in PROSPERO (CRD420251048860). Five electronic databases were searched for English-language studies published between 2015 and 2025. Studies were eligible if they involved undergraduate or postgraduate nursing students and evaluated AI, VR, HFS, or gamification in educational settings. Methodological quality was assessed using the Mixed Methods Appraisal Tool (MMAT 2018).

**Results:**

Forty studies met the inclusion criteria. HFS and VR were most frequently associated with improvements in knowledge, clinical judgment, and procedural skills. Gamification was associated with greater engagement, motivation, and knowledge retention. Evidence on AI was limited and preliminary, with a small number of studies suggesting possible benefits for critical thinking and problem-solving. Common barriers included technical problems, resource constraints, and inadequate faculty preparedness.

**Conclusions:**

TEL methods appear to improve student learning and provide safe environments for practice. Successful integration of TEL into nursing education requires institutional support, evidence-based teaching strategies, and continuous faculty development. TEL should complement rather than replace traditional educational practices. Future research should examine the long-term impact of TEL on learning outcomes, its cost-effectiveness, and its implementation in resource-constrained settings.

**Supplementary Information:**

The online version contains supplementary material available at https://doi.org/10.1186/s12909-026-10098-w.

## Background

The rapid pace of technological innovation, increasing complexity of patients, and increased expectations for digital competence in the present healthcare system require that nursing education utilize more agile and adaptable models of teaching [1, 2]. Traditional teaching methods that focus on content delivery and passive learning are not sufficient to prepare graduating nurses for the complexity of today’s clinical environments [3, 4].

Traditional clinical placements offer limited opportunities to interact with a range of different patients, variable levels of supervision from experienced clinical staff, and ethical concerns related to patient safety [5, 6]. This limits nursing students’ opportunities to develop critical thinking, clinical judgement, and confidence in their ability to make decisions based on evidence, which is crucial for providing safe and effective nursing care [7, 8].

In addressing these barriers, technology-enhanced learning (TEL) has emerged as an innovative approach to nursing education [9, 10]. TEL encompasses a range of digital technologies, including high-fidelity simulation (HFS), virtual reality (VR), gamification, and artificial intelligence (AI), which can help bridge the learning gap between the classroom and real-life clinical practice [11, 12].

These modalities foster active engagement, real-time decision-making, and reflective learning within safe, controlled environments. By providing opportunities for repeated practice, immediate feedback, and exposure to rare or complex clinical scenarios, TEL supports the development of competence and confidence while maintaining patient safety [13, 14].

High-fidelity simulation, in particular, has demonstrated consistent benefits in enhancing clinical judgment, critical thinking, self-efficacy, as well as reducing pre-clinical anxiety [15]. Similarly, VR and gamification provide immersive experiences that improve problem-solving, increase motivation, and enhance knowledge retention. Yıldız Çelik and Demiray found that VR-based simulation can surpass traditional methods in developing practical skills such as intravenous catheterization [16].

AI-driven platforms are increasingly acknowledged for delivering personalized learning, enhancing case-management performance, and supporting the development of higher-order thinking skills [3]. However, empirical evidence regarding the pedagogical effectiveness of AI remains limited, necessitating further research to clarify its educational value and ensure ethical and appropriate use [12].

While several reviews have examined individual technology-enhanced learning modalities in nursing education, few have systematically compared artificial intelligence, virtual reality, high-fidelity simulation, and gamification within a single synthesis. The novelty of this review lies in its cross-modality comparison, its incorporation of recent evidence published through 2025 including emerging AI applications, and its synthesis of implementation barriers and facilitators across modalities to inform practical decision-making in nursing curricula. By examining multiple TEL modalities together, this review provides broader insight into their relative educational value, implementation requirements, and remaining evidence gaps, including the limited evidence on long-term clinical performance outcomes [7, 13].

This systematic review aimed to synthesize current evidence on the educational outcomes associated with artificial intelligence, virtual reality, high-fidelity simulation, and gamification in nursing education, and to identify the factors that facilitate or hinder their implementation.

## Methods

### Study design

This systematic review was conducted in accordance with PRISMA (2020) guidelines. The protocol was prospectively registered in the International Prospective Register of Systematic Reviews (PROSPERO) under registration number CRD420251048860 in June 2025. No deviations from the protocol occurred.

### Research question

The review addressed the following question: What educational outcomes are associated with technology-enhanced learning methods, including artificial intelligence, virtual reality, high-fidelity simulation, and gamification, in nursing education, and what factors facilitate or hinder their implementation ?

### Search strategy

A comprehensive literature search was conducted in June 2025 across five major databases: PubMed, Cochrane Library, ScienceDirect, Web of Science, and Embase. The final search was last run on 5 July 2025.

The search strategy was guided by the PICO framework, where Population referred to nursing students, Intervention encompassed technology-enhanced learning methods (artificial intelligence, virtual reality, high-fidelity simulation, and gamification), Comparator represented traditional teaching approaches, and Outcomes included learning outcomes such as engagement, knowledge retention, confidence, skill acquisition, and clinical decision-making.

Search strategies were developed separately for each database using a combination of controlled vocabulary terms and free-text keywords. In PubMed, Medical Subject Headings (MeSH) were used to combine nursing education with each technology-enhanced learning modality. The PubMed search strings included: (“Education, Nursing“[Mesh]) AND (“Virtual Reality“[Mesh] OR “Video Games“[Mesh] OR “Artificial Intelligence“[Mesh] OR “Simulation Training“[Mesh]). Searches were limited to English-language studies because of resource and translation constraints. This restriction may have excluded relevant non-English evidence and is therefore addressed as a limitation. Additional filters applied included publication date within the last 10 years and free full text availability. The full database-specific search strategies are provided in supplementary material 1. Citation tracking and grey literature sources were not searched, which is also acknowledged as a limitation.

### Inclusion and exclusion criteria

#### Inclusion criteria

1. Studies involving undergraduate or postgraduate nursing students.
2. Studies evaluating AI, VR, HFS, or gamification, individually or in combination, within educational settings.
3. Studies measuring at least one relevant learning outcome: engagement, knowledge retention, clinical decision-making, reflective thinking, satisfaction, self-confidence, skill acquisition, or overall academic performance.
4. Randomized controlled trials (RCTs), quasi-experimental studies, quantitative, qualitative, or mixed methods designs.
5. Peer-reviewed publications in English from 2015 to 2025.

#### Exclusion criteria

1. Studies not focused on nursing students.
2. Studies focusing on technology use in clinical practice rather than educational settings.
3. Studies comparing only variations within the same technology (e.g., high- vs. low-fidelity simulation).
4. Conference abstracts, reviews, meta-analyses, editorials, essays, letters, commentaries, or non-English full texts.

### Study selection

All retrieved references were imported into Rayyan for deduplication and blinded screening. Two reviewers (AS and MM) independently screened titles and abstracts, followed by full-text review, using prespecified inclusion and exclusion criteria. Disagreements at either stage were resolved through discussion, with a third reviewer (MB) consulted to reach consensus where agreement could not be achieved. Formal interrater agreement statistics (e.g., Cohen’s kappa) were not calculated for the screening process; this is noted as a methodological limitation.

### Data extraction

A primary summary table was developed to present study characteristics and synthesis data, including author, year, country, study design, sample size, TEL modality, outcomes measured, main findings, and key study limitations. In response to the heterogeneity of outcome assessment across the included studies, a second summary table was developed to classify the outcome measurement approaches. This table extracted and categorized the type of instruments used as standardized/validated, researcher-developed/nonstandardized, or both. It also classified the reported outcomes as objective, subjective, or both.

### Quality appraisal

The methodological quality of the included studies was assessed using the Mixed Methods Appraisal Tool (MMAT 2018), which appraises study quality according to design-specific criteria. Each study was evaluated across five criteria and rated at the item level as “Yes”, “No”, or “Can’t tell”. Studies meeting four or five criteria were classified as high quality, those meeting three criteria as moderate quality, and those meeting two or fewer criteria as low quality. Two independent reviewers (MB and MM) conducted the appraisal, and disagreements were resolved through discussion and consensus. Detailed item-level MMAT ratings for all included studies are provided in supplementary material 2.

### Data synthesis

A narrative and thematic synthesis was conducted in response to heterogeneity in study designs and reported outcomes. Meta-analysis was not performed due to substantial variation in measurement approaches. The narrative synthesis summarized the impact of TEL strategies on nursing students’ learning outcomes, highlighting both similarities and differences in effectiveness across educational contexts. An inductive thematic approach was employed to identify recurrent patterns, barriers, and facilitators to implementation.

## Results

### Search outcomes

**Fig. 1:**
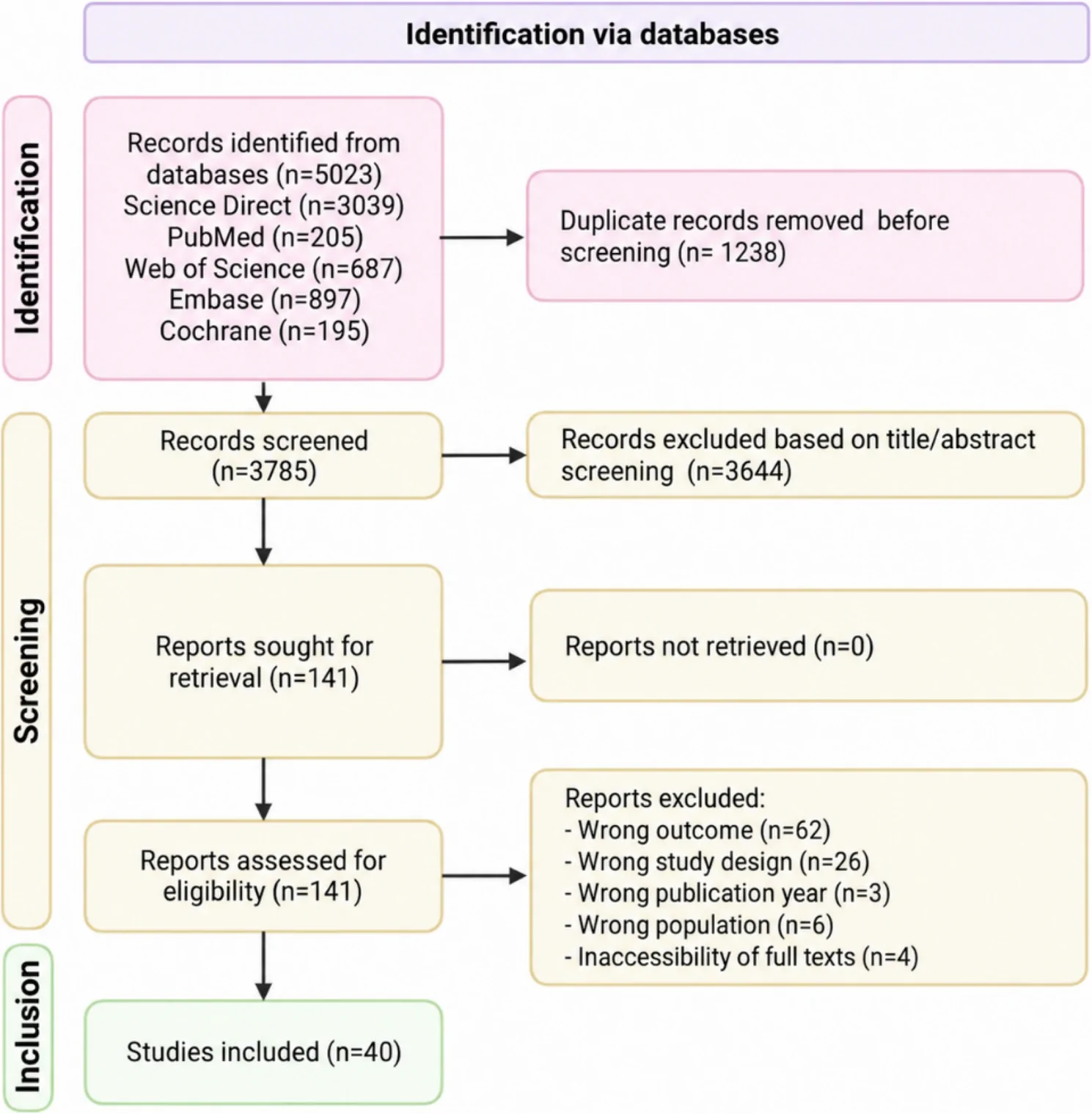
PRISMA flow diagram for the study selection process

The comprehensive search across 5 databases yielded a total of 5,023 articles. Following removal of 1,238 duplicate records, 3,785 unique titles and abstracts were screened. Of these, 3,644 were excluded, and 141 full-text reports were assessed for eligibility. At the full-text stage, 101 reports were excluded, primarily due to “Wrong outcome” (*n* = 62) and “Wrong study design” (*n* = 26). The remaining exclusions were due to “Wrong publication year” (*n* = 3), “Wrong population” (*n* = 6) and “Inaccessibility of full texts” (*n* = 4). Forty studies met all eligibility criteria and were included in the synthesis (Fig. 1).

All included studies were appraised using the Mixed Methods Appraisal Tool (MMAT 2018). Of the 40 included studies, 30 were rated *high quality*, 9 were rated *moderate quality*, and 1 was rated *low quality*. Despite the generally high-quality ratings, several methodological weaknesses recurred across the included studies. Small sample sizes were the most common limitation, with the majority of studies recruiting fewer than 100 participants, which constrains statistical power and the reliability of effect estimates. Single-site designs predominated, with most studies conducted at a single university or clinical training center, limiting transferability across different educational contexts and healthcare systems. Follow-up periods were typically short, usually restricted to an immediate post-intervention assessment, with few studies examining knowledge retention or skill durability beyond the intervention period. Outcome measurement relied heavily on self-reported instruments, particularly for confidence, satisfaction, and perceived competence, while objective performance indicators such as standardized skill checklists or instructor-rated assessments were used in a minority of studies. Several quasi-experimental designs also lacked adequate control conditions or blinding procedures, introducing risk of selection and performance bias.

### Characteristics of the included studies

The included studies were published between 2016 and 2025 and originated from 15 countries, with the largest number conducted in Turkey (*n* = 11) followed by Taiwan (*n* = 4), Spain (*n* = 4), the United States (*n* = 3), Palestine (*n* = 3), South Korea (*n* = 2), Saudi Arabia (*n* = 2), Lebanon (*n* = 2), Hong Kong (*n* = 2), United Kingdom (*n* = 2), Norway (*n* = 1), Germany (*n* = 1), China (*n* = 1), Denmark (*n* = 1) and Finland (*n* = 1).

The designs were heterogeneous and included randomized control trials (*n* = 15), quasi-experimental studies (*n* = 10), mixed-methods studies (*n* = 8), quantitative studies (*n* = 4), cross-sectional studies (*n* = 2) and qualitative studies (*n* = 1). The total sample size across all studies was 4,554 undergraduate and postgraduate nursing students.

High-Fidelity Simulation (HFS) studies were the most prevalent (*n* = 16), followed by Gamification (*n* = 10), Virtual Reality (VR) (*n* = 9), and Artificial Intelligence (AI) (*n* = 5).

The characteristics, main findings, and key limitations of the included studies are presented in Table 1.

**Table 1:** Summary of the included studies in the systematic review

Author, year, country	Type of study	Participants	Technology used	Outcome measured	Main findings	Key limitations
Lee et al. (2023) [17] Hong Kong	Randomized controlled trial	151 undergraduate nursing students	VR	Knowledge, self-confidence, satisfaction	Improved blood transfusion knowledge, self-confidence, and satisfaction	Single-site study, contamination risk, short-term follow-up, single-blind design
Tseng et al. (2020) [15] United States	Quantitative	90 undergraduate nursing students	HFS	Reflective thinking	Improved flexible and reflective thinking, debriefing was important	Small sample, self-report bias
Haddeland et al. (2021) [18] Norway	Randomized controlled trial	177 undergraduate nursing students	HFS	Knowledge, self-confidence	Improved knowledge, confidence, and knowledge retention	Variability in facilitation, limited long-term assessment, possible interview subjectivity
Yang et al. (2022) [19] South Korea	Quasi-experimental non-randomized controlled design	83 undergraduate nursing students	Gamification, VR	Knowledge, skills, self-confidence, problem-solving	Improved knowledge, problem-solving, self-confidence, and motivation	Short follow-up, self-reported outcomes, no objective competency measure, cognitive load with VR
Alkhalaf et al. (2022) [20] Saudi Arabia	Quantitative	68 undergraduate nursing students	HFS	Competency, skill transfer	Higher competency performance, but not statistically significant, better extravasation management	Small sample, implementation cost, stress/anxiety, possible instructor bias
Akalin et al. (2020) [21] Turkey	Mixed methods	107 undergraduate nursing students	HFS	Knowledge, critical thinking, clinical decision-making	Improved knowledge, critical thinking, and clinical decision-making	No long-term follow-up, limited qualitative sample, generalizability concerns
Plotzky et al. (2023) [22] Germany	Randomized mixed-methods study	131 undergraduate nursing students	VR	Knowledge acquisition, skill performance, satisfaction	VR improved satisfaction and realism, but not knowledge superiority, video performed better for some skills	Single-session exposure, observer subjectivity, technology-specific generalizability issues
Alsalamah et al. (2022) [23] Saudi Arabia Lebanon	Quantitative	673 undergraduate nursing students	HFS	Satisfaction, self-confidence, clinical judgment	High satisfaction and self-confidence, improved clinical judgment	Self-report bias, cultural/contextual differences
Chow et al. (2023) [24] Hong Kong	Mixed methods	183 undergraduate and 72 graduate nursing students	HFS	Self-Confidence, satisfaction, clinical decision-making	Improved confidence, clinical decision-making, and generic capabilities, reduced anxiety	Convenience sample, no control group, no post-training knowledge/skill test, technical issues
Molu (2025) [25] Turkey	Quasi-experimental	70 undergraduate nursing students	AI	Knowledge	AI-based care plans improved neonatal resuscitation learning	Small sample; self-report bias; single-center study, short-term follow-up, AI limitations
Chang et al. (2024) [26] Taiwan	Mixed methods	128 undergraduate nursing students	VR	Knowledge, learning motivation	Improved IV injection knowledge and motivation, strong learner acceptance	Single-site study, no control group, immediate post-test only, technical issues
Bailey et al. (2022) [27] United States	Quasi-experimental	26 undergraduate nursing students	HFS	Confidence	Increased nursing student confidence after simulation	Small sample, convenience sampling, no control group, narrow outcome focus
Gutiérrez-Puertas et al. (2021) [28] Spain	Randomized controlled trial	184 undergraduate nursing students	Gamification	Knowledge acquisition, knowledge retention Satisfaction	Improved knowledge retention and high user satisfaction	Convenience sample, no practical skill assessment, spanish-only instrument, limited generalizability
Arrogante et al. (2023) [29] Spain	Mixed methods	80 undergraduate nursing students	HFS	Communication skills, competency acquisition, knowledge retention	Improved competency, communication, psychological resources, and geriatric knowledge	Small sample, setting-specific findings, possible focus-group bias
Chang et al. (2024) [26] Taiwan	Quasi-experimental	50 undergraduate nursing students	AI	Critical thinking, problem-solving skills, overall learning	Generative AI improved critical thinking, problem-solving, and learning enjoyment	Small sample, limited process tracking, transition challenges from traditional teaching
Han et al. (2021) [30] China	Qualitative	9 undergraduate nursing students	Gamification	Competence, satisfaction	Positive attitudes, improved perceived competence, identified game-design challenges	Small sample, cultural specificity, recall bias, lack of standardized tools
Erdoǧan et al. (2023) [31] Turkey	Quasi-experimental	62 undergraduate nursing students	Gamification	Knowledge acquisition, knowledge retention	Improved knowledge and retention, positive attitudes toward digital games	Small sample, self-selection bias, single institution, short duration
Dragnes Brix et al. (2025) [32] Denmark	Mixed methods	59 undergraduate nursing students	VR, HFS	Self-efficacy Competence Confidence	VR before HFS improved self-efficacy, competence, and confidence	Self-reported outcomes, small sample, no long-term data, VR discomfort/cost
Jeong et al. (2024) [33] South Korea	Randomized controlled trial	59 undergraduate nursing students	VR	Knowledge, satisfaction, self-efficacy, confidence	IVR improved knowledge, satisfaction, self-efficacy, and confidence	Single university, technical limitations, narrow practicum focus
Bayram et al. (2019) [34] Turkey	Randomized controlled trial	86 undergraduate nursing students	VR, gamification	Skills, knowledge	Improved tracheostomy skill performance, knowledge differences were limited	Short intervention, limited monitoring
Singleton et al. (2022) [35] UK	Randomized controlled trial	171 undergraduate nursing students	VR	Knowledge acquisition, engagement, confidence	Improved hypoglycemia knowledge and engagement, immersive learning pathway identified	Short-term knowledge measurement, narrow topic scope, self-report bias, limited diversity
Ayed et al. (2023) [36] Palestine	Randomized controlled trial	150 undergraduate nursing students	HFS	Clinical decision-making	Significantly improved clinical decision-making	Self-report bias, no blinding, single institution, limited clinical exposure context
Hodrob et al. (2024) [37] Palestine	Randomized controlled trial	154 undergraduate nursing students	HFS	Skills, satisfaction, self-confidence	Improved airway management performance, satisfaction, and self-confidence	Single-site study, limited performance measurement, self-report bias, controlled setting may limit realism
Sanz-Martos et al. (2024) [38] Spain	Quasi-experimental	122 undergraduate nursing students	Gamification	Satisfaction, knowledge, critical thinking	Improved satisfaction, knowledge, and critical thinking	Content differences between groups, possible overestimation of satisfaction, implementation challenges
Lee et al. (2024) [39] South Korea	Randomized controlled trial	66 undergraduate nursing students	VR	Knowledge, confidence, clinical reasoning	Increased immersion and satisfaction, but no significant gains in knowledge, confidence, or clinical reasoning	Single session, limited generalizability, sensitivity of measures, prior ECMO exposure may affect results
Ayed et al. (2022) [40] Palestine	Quasi-experimental	150 undergraduate nursing students	HFS	Clinical judgment	Improved clinical judgment in pediatric nursing students	Short duration, single scenario, convenience sample, single university
Adhikari et al. (2021) [41] UK	Mixed methods	19 undergraduate nursing students	VR	Self-Efficacy, confidence, clinical decision making	IVR sepsis game improved self-confidence and reduced anxiety, qualitative acceptability themes identified	Small sample, recruitment challenges, technology comfort bias, single time-point measurement
Akutay et al. (2024) [42] Turkey	Randomized controlled trial	188 undergraduate nursing students	AI	Case management performance, satisfaction, knowledge	AI-supported cases improved case management performance, satisfaction similar to control	Generalizability limits, feedback/interaction constraints, no long-term retention assessment
Vural Doğru et al. (2020) [43] Turkey	Randomized controlled trial	72 undergraduate nursing students	HFS	Knowledge, skills	HFS outperformed traditional teaching for knowledge and skills	Small sample, single-center study
Peng et al. (2024) [44] Taiwan	Randomized controlled trial	84 undergraduate nursing students	HFS	Knowledge, skills, critical thinking, self-confidence	Improved intubation, PPE, ECG performance, critical thinking, and self-confidence	Small sample, limited real-world replication, resource requirements
Onarlcl et al. (2021) [45] Turkey	Randomized controlled trial	61 undergraduate nursing students	HFS	Knowledge, satisfaction, confidence	Improved burn-care knowledge, satisfaction, and self-confidence	Incomplete separation of intervention and control groups
Gonzalez-Garcia et al. (2025) [46] Spain	Cross-sectional	86 undergraduate nursing students	AI	Academic performance, engagement	ChatGPT use is associated with improved grades and perceived learning support	Self-report bias, limited generalizability, weak correlation strength
Öz et al. (2021) [47] Turkey	Quasi-experimental	110 undergraduate nursing students	Gamification	Knowledge, skills	Improved knowledge and skill performance	Generalizability limits, possible contribution of video support
Koivisto et al. (2016) [48] Finland	Cross-sectional	166 undergraduate nursing students	Gamification	Knowledge, clinical reasoning	Improved information gathering, decision-making, and clinical reasoning	Instrument validity concerns, limited generalizability
Avşar et al. (2023) [49] Turkey	Quasi-experimental	95 undergraduate nursing students	Gamification	Learning outcomes	Significant difference favoring gamified learning	Non-randomized groups, short-term assessment, limited generalizability
Ambrosio Mawhirter et al. (2016) [50] USA	Quantitative	18 undergraduate nursing students	Gamification	Engagement, motivation	High engagement and increased readiness for clinical practice	Small sample, limited generalizability
Kudubes (2025) [51] Turkey	Randomized controlled trial	60 undergraduate nursing students	Gamification	Knowledge, motivation	Kahoot group showed higher motivation and better exam performance	Limited generalizability
Fawaz et al. (2016) [52] Lebanon	Quasi-experimental	56 undergraduate nursing students	HFS	Clinical judgement, motivation	Improved clinical judgment and motivation	Limited generalizability
Cengiz et al. (2023) [53] Turkey	Randomized controlled trial	112 undergraduate nursing students	HFS	Self-efficacy, problem solving	Improved self-efficacy and problem-solving	Lack of objective competency assessment, limited generalizability
Hwang et al. (2025) [54] Taiwan	Mixed methods	56 undergraduate nursing students	AI	Critical thinking, learning outcomes	GenAI-guided prompts improved critical thinking, metacognition, problem-solving, and learning outcomes	Small sample, need for randomized control trial

### Outcome measurement characteristics

Across the 40 included studies, outcome measurement approaches varied considerably. Thirteen studies used only standardized or previously validated instruments, 14 used only researcher-developed or nonstandardized instruments, and 13 used a combination of both.

Regarding outcome type, 6 studies assessed objective outcomes only, 11 assessed subjective outcomes only, and 23 assessed both objective and subjective outcomes. Objective outcomes commonly included knowledge test scores, examination grades, OSCE scores, procedural skill checklists, academic performance indicators, care plan scores, and externally assessed performance rubrics. Subjective outcomes included self-confidence, satisfaction, motivation, anxiety, self-efficacy, perceived learning, engagement, technology acceptance, reflective thinking, flexible thinking, and qualitative learning experiences.

The summary of outcome measurement approaches in the included studies is presented in Table 2.

**Table 2:** Summary of outcome measurement approaches in the included studies

Author, year, country	Technology used	Outcome type	Instrument type	Objective outcome	Subjective outcome
Lee et al. (2023) [17] Hong Kong	VR	Both objective and subjective outcomes	Standardized/validated instruments	Blood transfusion knowledge	Satisfaction, self-confidence
Tseng et al. (2020) [15] United States	HFS	Subjective outcomes only	Standardized/validated instruments	None	Reflective thinking
Haddeland et al. (2021) [18] Norway	HFS	Both objective and subjective outcomes	Researcher-developed / nonstandardized instruments	Knowledge test	Self-confidence
Yang et al. (2022) [19] South Korea	Gamification VR	Both objective and subjective outcomes	Standardized/validated instruments	Neonatal resuscitation knowledge	Problem-solving ability, clinical reasoning ability, self-confidence, anxiety, learning motivation
Alkhalaf et al. (2022) [20] Saudi Arabia	HFS	Objective outcomes only	Standardized/validated instruments	Extravasation chemotherapy management competency, skill transfer	None
Akalin et al. (2020) [21] Turkey	HFS	Both objective and subjective outcomes	Both validated and researcher-developed instruments	Knowledge assessment form	Critical thinking disposition, clinical decision-making perception
Plotzky et al. (2023) [22] Germany	VR	Both objective and subjective outcomes	Both standardized/validated and researcher-developed instruments	Endotracheal suctioning knowledge, OSCE skill performance	Learner satisfaction, VR acceptance, qualitative learning experience
Alsalamah et al. (2022) [23] Saudi Arabia Lebanon	HFS	Subjective outcomes only	Standardized/validated instruments	None	Satisfaction, self-confidence, clinical judgment
Chow et al. (2023) [24] Hong Kong	HFS	Subjective outcomes only	Both standardized/validated and researcher-developed instruments	None	Generic capabilities, self-confidence, satisfaction
Molu (2025) [25] Turkey	AI	Both objective and subjective outcomes	Researcher-developed / nonstandardized instruments	Neonatal resuscitation knowledge test	Perceptions of AI applications in nursing
Chang et al. (2024) [26] Taiwan	VR	Both objective and subjective outcomes	Both standardized/validated and researcher-developed instruments	Intravenous injection procedural knowledge	VR acceptance, learning motivation
Bailey et al. (2022) [27] United States	HFS	Subjective outcomes only	Standardized/validated instruments	None	Confidence
Gutiérrez-Puertas et al. (2021) [28] Spain	Gamification	Both objective and subjective outcomes	Both standardized/validated and researcher-developed instruments	Knowledge acquisition and retention test	Satisfaction
Arrogante et al. (2023) [29] Spain	HFS	Both objective and subjective outcomes	Researcher-developed / nonvalidated instruments	Nursing competency performance using verification list	Student perceptions from focus groups
Chang et al. (2024) [26] Taiwan	AI	Subjective outcomes only	Standardized/validated instruments	None	Critical thinking, problem solving, learning enjoyment
Han et al. (2021) [30] China	Gamification	Subjective outcomes only	Researcher-developed / nonstandardized instruments	None	Perceived competence improvement, satisfaction
Erdoǧan et al. (2023) [31] Turkey	Gamification	Objective outcomes only	Researcher-developed / nonstandardized instruments	Developmental care knowledge test, retention knowledge test	None
Dragnes Brix et al. (2025) [32] Denmark	VR HFS	Subjective outcomes only	Researcher-developed / nonvalidated instruments	None	Self-efficacy, professional competence, confidence, student perceptions
Jeong et al. (2024) [33] South Korea	VR	Both objective and subjective outcomes	Standardized/validated instruments	Knowledge of normal vaginal delivery care	Satisfaction, self-efficacy, confidence
Bayram et al. (2019) [34] Turkey	VR gamification	Objective outcomes only	Researcher-developed / nonvalidated instruments	Knowledge test, OSCE-based tracheostomy care skill performance	None
Singleton et al. (2022) [35] UK	VR	Both objective and subjective outcomes	Both standardized/validated and researcher-developed instruments	Hypoglycemia knowledge test	Engagement, confidence
Ayed et al. (2023) [36] Palestine	HFS	Subjective outcomes only	Standardized/validated instruments	None	Perceived clinical decision-making
Hodrob et al. (2024) [37] Palestine	HFS	Both objective and subjective outcomes	Standardized/validated instruments	Airway management performance checklist	Satisfaction, self-confidence
Sanz-Martos et al. (2024) [38] Spain	Gamification	Both objective and subjective outcomes	Researcher-developed / nonstandardized instruments	Knowledge test score, practical activity checklist scores	Satisfaction, perceived usefulness
Lee et al. (2024) [39] South Korea	VR	Both objective and subjective outcomes	Both standardized/validated and researcher-developed instruments	ECMO nursing knowledge test	Confidence, clinical reasoning capacity
Ayed et al. (2022) [40] Palestine	HFS	Objective outcomes only	Standardized/validated instrument	Clinical judgment assessed using Lasater Clinical Judgment Rubric	None
Adhikari et al. (2021) [41] UK	VR	Subjective outcomes only	Both validated and researcher-developed instruments	None	Self-confidence, perceived knowledge, perceived decision-making
Akutay et al. (2024) [42] Turkey	AI	Both objective and subjective outcomes	Researcher-developed / nonstandardized instruments	Knowledge test, priority nursing diagnoses/case performance	Satisfaction
Vural Doğru et al. (2020) [43] Turkey	HFS	Both objective and subjective outcomes	Both standardized/validated and researcher-developed instruments	Cardiac auscultation knowledge test, OSCE-based cardiac auscultation skill assessment	Anxiety
Peng et al. (2024) [44] Taiwan	HFS	Both objective and subjective outcomes	Both standardized/validated and researcher-developed instruments	Emergency/critical care knowledge, OSCE skills for intubation, PPE, and 12-lead ECG	Self-confidence, critical thinking
Onarlcl et al. (2021) [45] Turkey	HFS	Both objective and subjective outcomes	Both standardized/validated and researcher-developed instruments	Burn patient care knowledge, burn patient care plan score	Satisfaction, self-confidence
Gonzalez-Garcia et al. (2025) [46] Spain	AI	Both objective and subjective outcomes	Researcher-developed / nonstandardized instruments	Academic performance/GPA	Perceived usefulness, perceived academic improvement, satisfaction, opinions on ChatGPT
Öz et al. (2021) [47] Turkey	Gamification	Objective outcomes only	Researcher-developed / nonstandardized instruments	Knowledge test, observed IM injection skill checklist	None
Koivisto et al. (2016) [48] Finland	Gamification	Subjective outcomes only	Researcher-developed / nonstandardized instruments	None	Perceived learning of clinical reasoning, application of nursing knowledge
Avşar et al. (2023) [49] Turkey	Gamification	Objective outcomes only	Researcher-developed / nonvalidated instruments	Achievement test score	None
Ambrosio Mawhirter et al. (2016) [50] USA	Gamification	Both objective and subjective outcomes	Researcher-developed / nonstandardized instruments	Simulation rubric scores for patient safety, environment of care, communication, and emergency response	Perceived readiness, motivation
Kudubes (2025) [51] Turkey	Gamification	Both objective and subjective outcomes	Both standardized/validated and researcher-developed instruments	Midterm, final, and overall exam grade	Motivation
Fawaz et al. (2016) [52] Lebanon	HFS	Both objective and subjective outcomes	Standardized/validated instruments	Clinical judgment	Motivation
Cengiz et al. (2023) [53] Turkey	HFS	Subjective outcomes only	Standardized/validated instruments	None	Self-efficacy, problem-solving skills
Hwang et al. (2025) [54] Taiwan	AI	Both objective and subjective outcomes	Both standardized/validated and researcher-developed instruments	Question-generation via prompt performance	Critical thinking tendency, metacognition tendency, problem-solving tendency

### Learning outcomes

#### Knowledge acquisition and retention

Knowledge-related outcomes were assessed in 28 studies, and most reported improvements following TEL interventions. VR was associated with higher post-test knowledge scores and better conceptual understanding than traditional lectures as reported by J. J. Lee et al. [17], Singleton et al. [35],Yang & Oh [19], and H. Lee et al. [39]. However, H. Lee et al. [39] emphasized that single-session VR exposure resulted in only modest gains in knowledge without repeated practice [17, 19, 35, 39] .

High-fidelity simulation was also associated with improved knowledge acquisition and recall, with studies from Norway, Turkey, and Taiwan reporting that repeated simulation exposure supported students’ consolidation and application of theoretical knowledge in clinical contexts [18, 43, 44]. Gamification was linked to both immediate and long-term knowledge retention, with higher post-test and retention scores reported in several studies [28, 31, 38, 47, 49].

Evidence on AI-supported learning was more limited, but the included studies suggested potential improvements in conceptual understanding in neonatal resuscitation, case-management performance, and higher-order thinking [25, 26, 55].

#### Clinical skill development and performance

Skill-related outcomes were assessed in 24 studies. HFS was the modality most frequently associated with improved procedural and applied clinical performance, including airway management, intravenous therapy, clinical decision-making, intubation, personal protective equipment (PPE) use, and electrocardiogram (ECG) interpretation [18, 27, 36, 37, 44].

Gamification and VR also supported skill development in several studies. Yang & Oh reported improved psychomotor coordination and problem-solving abilities following gamified VR training, while Bayram & Caliskan found greater procedural accuracy in tracheostomy care among learners exposed to game-based interventions [19, 34]. Plotzky et al. reported greater realism and learner satisfaction in the VR group, although performance outcomes were inconsistent when compared to video-based training [22]. Akutay et al. found improved case-management performance among learners supported by AI, whereas Hwang et al. [54] reported gains in problem-solving tendencies and metacognitive skills; however, these findings remain preliminary [33, 42].

#### Self-confidence, self-efficacy, and satisfaction

VR was associated with increased confidence and satisfaction in multiple studies, including J. J. Lee et al., Jeong & Cha [33], and Adhikari et al. [17, 23, 41]. HFS was also linked to higher self-confidence before clinical placements, Alsalamah et al. [23], Hodrob et al. and Ayed et al. reported gains in both confidence and decision-making [30, 36, 37]. Gamification has been shown to improve perceived competence [21, 28, 51]. AI-based platforms showed preliminary positive trends, particularly in confidence in reasoning and case-management skills, suggesting possible benefits of individualized feedback and adaptive guidance [25, 26]. However, most of these outcomes were based on self-reported measures.

#### Critical thinking, problem-solving, and clinical judgment

Sixteen studies assessed higher-order cognitive outcomes, including critical thinking, problem-solving, reflective thinking, and clinical judgment. AI-based approaches provided preliminary evidence of potential benefits on critical thinking, metacognition, and problem-solving [26]. HFS was also associated with improvements in reflective thinking, adaptability, and clinical judgment [15, 24, 29, 36, 40]. VR and gamified simulations supported diagnostic reasoning and adaptability [19]. Arrogante et al. [29] additionally reported improved communication and teamwork in mixed-methods simulation activities [48].

#### Engagement and motivation

Engagement and motivation were evaluated in 19 studies, with most reporting more positive learner engagement in TEL modalities than traditional instruction. Gamification showed the most consistent effects, with increased focus, enjoyment, and motivation, as reported by Koivisto et al. [48], Avşar et al., Sanz-Martos et al., and Ambrosio Mawhirter & Ford Garofalo [20, 38, 49, 50]. Some studies also reported reduced preclinical anxiety in game-based learning contexts.

VR was associated with strong learner engagement through immersive and multisensory experiences, although some participants reported cognitive fatigue after extended sessions [19, 22, 35]. AI-based learning systems suggested potential benefits for learner motivation and personalized feedback, but evidence for sustained effects remains limited [26, 54].

#### Barriers and facilitators to implementation

Several studies reported implementation-related barriers. Common challenges included software instability, hardware malfunctions, high cost of VR headsets or simulation equipment, and limited on-site technical support, particularly in VR- and HFS-based interventions [22, 23, 29]. Faculty readiness was also identified as a concern, with some studies highlighting the need for additional instructor training to integrate VR, AI, or simulation technologies into teaching, especially in settings with limited access to simulation laboratories or digital infrastructure [18, 56]. Other reported barriers included short intervention durations, limited sample size, usability issues, physical discomfort associated with head-mounted devices, and the time required for students to adapt to unfamiliar digital tools [25, 34].

Reported facilitators were less frequently described, but several factors supported implementation across studies. These included structured debriefing and guided reflection in simulation-based learning, repeated practice opportunities, clear learning objectives, safe learning environments, and positive learner acceptability [15, 18]. Some studies also reported high levels of learner satisfaction and favorable attitudes toward TEL, particularly in gamified and simulation-based interventions, which may support uptake in educational settings [23, 28, 30]. In addition, realism, immersion, and opportunities to apply theoretical knowledge in clinically relevant scenarios were reported as features that appeared to support learner engagement and implementation in some VR- and HFS-based interventions [22, 40, 44].

## Discussion

This systematic review aimed to synthesize current evidence on the educational outcomes associated with technology-enhanced learning (TEL), including high-fidelity simulation (HFS), virtual reality (VR), gamification, and artificial intelligence (AI), in nursing education, and to identify the factors that facilitate or hinder their implementation.

Overall, the included studies suggested that TEL was associated with improvements across several domains, including knowledge, clinical and procedural skills, confidence, engagement, and selected higher-order cognitive outcomes. These findings align with earlier reviews reporting that digital and immersive strategies can strengthen cognitive, psychomotor, and affective learning outcomes among nursing students [57–59].

Among the included modalities, HFS showed the most consistent pattern of positive findings, particularly in relation to knowledge, procedural performance, clinical judgment, and learner confidence. VR also demonstrated educational value, especially for engagement, satisfaction, and experiential learning, although its effects on knowledge and performance were less consistent. Gamification was most strongly associated with motivation, engagement, and knowledge retention, consistent with previous findings on game-based learning in nursing education [59, 60]. AI showed emerging promise for critical thinking, problem-solving, case-based reasoning, and personalized learning support, in line with recent reviews of AI [61, 62]. However, the AI evidence base in this review was smaller and more recent than the evidence for HFS, VR, or gamification, and most AI studies were limited by short duration and small sample size. These findings should therefore be interpreted cautiously. In addition, ethical and practical concerns, including transparency, data privacy, and the potential for bias, remain important considerations for implementation in nursing education [63].

The interpretation of these findings should also consider the outcome measurement approaches used across the included studies. Although several studies reported objective outcomes such as knowledge tests, examination grades, OSCE-based performance scores, procedural checklists, care plan scores, and externally assessed performance rubrics, subjective outcomes were also frequently reported. These included self-confidence, satisfaction, motivation, perceived learning, self-efficacy, technology acceptance, anxiety, engagement, and qualitative learning experiences. Therefore, improvements in learner satisfaction, confidence, or motivation should not be interpreted as direct evidence of improved clinical competence unless supported by objective performance measures. This distinction is particularly important in TEL research, where positive learner perceptions may reflect acceptability and engagement rather than measurable improvement in clinical skill performance.

For educators considering the adoption of technology-enhanced learning, the findings suggest that the choice of modality should be guided by the intended learning outcome, available resources, and faculty readiness. High-fidelity simulation may be most suitable when the aim is to strengthen procedural skills, clinical judgment, teamwork, and decision-making in a safe environment, particularly when structured debriefing and repeated practice are feasible. Virtual reality may be useful for immersive exposure to rare, complex, or high-risk clinical scenarios, but its adoption requires attention to equipment cost, technical support, learner comfort, and integration with clear learning objectives. Gamification may be appropriate when the goal is to increase motivation, engagement, formative assessment, and knowledge retention, especially in theoretical or skills-preparation courses. Artificial intelligence-supported learning may offer opportunities for personalized feedback, case-based reasoning, and problem-solving; however, educators should adopt AI cautiously because the current evidence remains limited and issues related to accuracy, transparency, academic integrity, data privacy, and faculty oversight require careful consideration [18, 36, 40].

Implementation challenges were reported frequently across the included studies. Common barriers included high equipment costs, limited hardware access, software instability, inadequate technical support, and insufficient faculty preparedness. These barriers are consistent with previous reviews that identified cost, infrastructure, and educator readiness as important constraints in TEL adoption [64, 65]. Facilitators were described less often, but positive learner acceptability, clear learning objectives, repeated practice, and structured debriefing appeared to support implementation across several studies. These findings suggest that TEL is most effective when embedded within pedagogically structured learning environments rather than introduced as technology alone.

The strength of evidence varies across modalities and should be interpreted in light of the methodological profile of the supporting studies. HFS demonstrated the most consistent findings, but most studies were single-site with self-reported primary outcomes. VR findings were positive but largely based on single-session interventions with immediate post-test measurement [66, 67]. Gamification evidence was consistent for engagement and motivation but relied predominantly on researcher-developed instruments and short assessment windows. AI evidence was the most preliminary, comprising a small number of methodologically limited studies with no long-term follow-up. Taken together, these limitations mean that while TEL shows consistent promise across outcome domains, the magnitude and durability of these effects cannot yet be established with confidence, and findings should be interpreted accordingly.

The findings support the use of TEL as a complement to, rather than a replacement for traditional nursing education. This is consistent with previous research emphasizing that technology should support, rather than replace the human elements of teaching (8,63). Future research should move beyond short-term, single-center evaluations and focus on multi-center and longitudinal study designs, objective performance outcomes, cost-effectiveness, and implementation across diverse educational settings, particularly in low-resource contexts. Further research is also needed to clarify the pedagogical and ethical implications of AI-supported learning in nursing education.

### Limitations

Several limitations are present in this review. First, the search was restricted to studies published in English between 2015 and 2025 and available as full text, which may have excluded relevant evidence published in other languages or unavailable through full-text access and introduced language, availability, and time-frame bias. In addition, although multiple databases were searched, the search strategy used modality-specific combinations of nursing education and technology-related terms, and the syntax was adapted across databases according to platform functionality. As a result, some relevant studies using alternative terminology may not have been retrieved. Grey literature sources and citation tracking were not searched, which may have led to the omission of unpublished studies, conference papers, dissertations, or additional relevant studies.

Second, the included studies showed substantial heterogeneity in design, sample size, intervention characteristics, and outcome measures. This limited comparability across studies and prevented quantitative meta-analysis. Outcome measurement also varied considerably. Several studies used researcher-developed or intervention-specific instruments, which may limit comparability and measurement consistency across studies. Many of the reported outcomes, particularly confidence, satisfaction, and perceived competence, were based on self-reported measures rather than objective performance indicators. Therefore, positive findings related to learner perceptions should be interpreted cautiously when not supported by objective outcomes such as knowledge tests, OSCE-based performance scores, procedural checklists, or externally assessed clinical performance.

Third, although most studies were classified as moderate to high quality, many were conducted at single institutions and used short follow-up periods, which restricts generalizability and limits understanding of the long-term durability of learning gains. In addition, formal interrater agreement statistics, such as Cohen’s kappa, were not calculated during the screening process, which should be considered a methodological limitation.

Finally, publication bias may be present, because studies with positive findings are more likely to be published in peer-reviewed journals. A formal publication bias assessment was not conducted because of the heterogeneity of study designs, interventions, and outcomes. Furthermore, the certainty of evidence was not graded using GRADE or a comparable framework. Therefore, the conclusions of this review should be interpreted in relation to the methodological quality, heterogeneity, and measurement limitations of the included studies.

## Conclusion

Technology-enhanced learning has become an increasingly important component of nursing education. Across the included studies, HFS, VR, gamification, and AI were associated with improvements in knowledge, selected clinical skills, confidence, engagement, and higher-order cognitive outcomes. However, the educational value of these approaches depended on how they were implemented. Structured instructional design, repeated practice, debriefing, faculty preparedness, and institutional support emerged as important enabling factors, whereas technical problems, high resource demands, and limited infrastructure remained common barriers. TEL should therefore be integrated as a complement to, rather than a replacement for traditional nursing education. Future research should prioritize multi-center and longitudinal designs, examine cost-effectiveness, and evaluate implementation across diverse and resource-constrained educational settings.

## Supplementary Information

Supplementary Material 1.

Supplementary Material 2.

## Data Availability

All data generated or analysed during this study are included in this published article and its supplementary information files.

## Abbreviations

TEL: Technology-enhanced learning
AI: Artificial intelligence
VR: Virtual reality
HFS: High-fidelity simulation
MeSH: Medical Subject Headings
RCT: Randomized-control trial
OSCE: Objective Structured Clinical Examination
PPE: Personal protective equipment
ECG: Electrocardiogram
IM: Intra-muscular
