# Supplementary material for: Technology-enhanced learning in nursing education: a systematic review
Source: BMC Med Educ. 2026 Aug 11;26:1802. doi: 10.1186/s12909-026-10098-w (PMC13741648; doi:10.1186/s12909-026-10098-w)
Supplement: Supplementary file 1 [file 12909_2026_10098_MOESM1_ESM.docx]

Search strategies used in each database

**PubMed**
Filters applied: Publication date: 2015–2025; English; free full text
Date last run: 5 July 2025

1. ("Education, Nursing"[Mesh]) AND "Virtual Reality"[Mesh]
2. ("Education, Nursing"[Mesh]) AND "High Fidelity Simulation Training"[Mesh]
3. ("Education, Nursing"[Mesh]) AND "Artificial Intelligence"[Mesh]
4. ("Education, Nursing"[Mesh]) AND "Gamification"[Mesh]

**ScienceDirect**
Filters applied: Publication year: 2015–2025; English; open access
Date last run: 5 July 2025

1. ("nursing education") AND ("artificial intelligence")
2. ("nursing education") AND ("gamification")
3. ("nursing education") AND ("virtual reality")
4. ("nursing education") AND ("high-fidelity simulation")

**Embase**
Filters applied: Publication year: 2015–2025; English
Date last run: 5 July 2025

1. ('nursing education'/exp OR 'nursing education') AND ('artificial intelligence'/exp OR 'artificial intelligence')
2. ('nursing education'/exp OR 'nursing education') AND ('virtual reality'/exp OR 'virtual reality')
3. ('nursing education'/exp OR 'nursing education') AND ('high fidelity simulation training'/exp OR 'high fidelity simulation training')
4. ('nursing education'/exp OR 'nursing education') AND ('gamification'/exp OR 'gamification')

**Cochrane Library**
Filters applied: Publication year: 2015–2025
Date last run: 5 July 2025

1. ("nursing education"):ti,ab,kw AND ("artificial intelligence"):ti,ab,kw
2. ("nursing education"):ti,ab,kw AND ("virtual reality"):ti,ab,kw
3. ("nursing education"):ti,ab,kw AND ("high-fidelity simulation" OR "high fidelity simulation training"):ti,ab,kw
4. ("nursing education"):ti,ab,kw AND ("gamification"):ti,ab,kw

**Web of Science**
Filters applied: Publication year: 2015–2025; English; document type: article or review
Date last run: 5 July 2025

1. ("virtual reality" OR "video games" OR "artificial intelligence" OR "simulation training") AND ("nursing education")
